# *Leymus chinensis* Adapts to Degraded Soil Environments by Changing Metabolic Pathways and Root Exudate Components

**DOI:** 10.3389/fpls.2022.894346

**Published:** 2022-05-26

**Authors:** Yulong Lin, Pan Zhang, Qingying Wu, Ying Zhang, Qianhao Wei, Yihang Sun, Yuchen Wu, Shixuan Sun, Guowen Cui

**Affiliations:** ^1^School of Animal Science and Technology, Northeast Agricultural University, Harbin, China; ^2^School of Resources and Environment, Northeast Agricultural University, Harbin, China

**Keywords:** phytoremediation, metabolomics, root exudates (RE), soil degradation, feed crop

## Abstract

Phytoremediation is a promising remediation strategy for degraded soil restoration. Root exudates are the main carrier substances for information communication and energy transfer between plant roots and soil, which play non-negligible roles in the restoration process. This work investigated the adaptation of *Leymus chinensis* root exudates to different degraded levels of soil and the mechanism of rhizosphere restoration in a 3-year degraded soil field study. We found that the soil quality at each degradation level significantly increased, with the soil organic matter (SOM) content slightly increasing by 1.82%, moderately increasing by 3.27%, and severely increasing by 3.59%, and there were significant increases in the contents of available nutrients such as available phosphorus (AP), ammonia nitrogen (AN), and nitrate nitrogen (NN). The physiological activities indicated that root tissue cells also mobilize oxidative stress to respond to the soil environment pressure. A total of 473 main components were obtained from root exudates by gas chromatography–time-of-flight mass spectrometry (GC–TOFMS), including acids, alcohols, carbohydrates, and other major primary metabolites. OPLS-DA revealed that soil degradation exerted an important influence on the metabolic characteristics of root exudates, and the numbers of both up- and downregulated metabolic characteristic peaks increased with the increase in the degree of degradation. Forty-three metabolites underwent clear changes, including some defense-related metabolites and osmotic adjustment substances that were significantly changed. These changes mainly mobilized a series of lipid metabolism pathways to maintain the fluidity of membrane function and help plants adapt to unfavorable soil environmental conditions. The PPP energy metabolism pathway was mobilized in response to slight degradation, and TCA energy pathways responded to the environmental pressure of severe soil degradation.

## Introduction

Soil degradation leads to salinization, soil organic matter (SOM) loss, decreased fertility, unbalanced elements, reduced aggregate stability, and deterioration of soil structure (Tamene et al., 2019; Jin et al., 2022). It has severely affected crop growth and economic productivity and has become a worldwide disaster. Phytoremediation is an environmentally friendly, economical, and effective method to remediate degraded soils (Liu et al., 2018). As an important microhabitat surrounding the plant root surface, the rhizosphere links the bulk soil and living roots, which are generally involved in the phytoremediation process. In the local area, root exudates play an important role in the soil remediation process (Zhang et al., 2021).

Roots can continuously secrete a large amount of root exudates into the rhizosphere during plant growth. These exudates are mainly composed of organic acids, amino acids, lipids, phenols, alcohols, and other low-molecular-weight organic metabolites, high-molecular-weight substances such as mucus and protein, and other special metabolites (Rohrbacher and St-Arnaud, 2016). Although the biological functions of these compounds have not been confirmed, some low-molecular-weight organic compounds (LMWOA) have been considered carrier substances for energy transfer media and communication signals between plants, soil, and rhizosphere microorganisms (Hu et al., 2018). The main components and abundance of root exudates vary with plant species, environmental conditions, soil nutrients, soil types, and other factors. Plants will adjust their nutrition, cells, physiology, and biochemistry when encountering extreme soil environments and enhance their adaptability to reduce the negative effects of adverse environmental conditions (Rodriguez-Garrido et al., 2020). For example, plants increase the root exudate rate by altering root morphological characteristics and associated changes in underground C allocation to cope with elevated temperatures (Yin et al., 2013). Shen et al. (2020) studied how plants respond to long-term overgrazing soil degradation by enhancing species-specific enhancements to the root exudation rate and changing the formation and stability of grassland SOC by releasing root exudates.

It is important to explore the ecological and physiological responses and mechanisms of plant root exudates in the process of soil restoration. Recently, different scholars have conducted research on root exudates and their ecological applications. A few studies have found that the intercropping system of *Moso bamboo* and *Sedum plumbizincicola* increased the organic acids in root exudates and facilitated the absorption of heavy metals by *Moso bamboo* (Bian et al., 2021). Peizataifeng rice increased the contents of oxalic acid, critic acid, and malonic acid in root exudates and promoted DBP and DEHP desorption in the soil (Du et al., 2020). *Sonchus asper* L. and *Vicia faba* L. have been reported to adapt to cadmium stress by regulating LMWOA in root exudates (Zhan et al., 2016). However, current phytoremediation research mainly focuses on pollutants and heavy metals, and the actual *in situ* soil rhizosphere process has not been fully demonstrated beyond idealized laboratory research. There have been few studies on the total components of plant root exudate analysis and differential metabolite identification. The composition of metabolites secreted by plant roots in degraded soil environments has changed, which indicates that the metabolic pathways involved have also changed. There have been few studies on the *Leymus chinensis* root exudate characteristics and metabolic pathways in degraded soil restoration. The emergence and development of metabolomics provide new ideas for this research. Metabolomics is derived from systems biology and identifies significant differences in key biomarkers and has been widely used in defining the tolerance response of plants under external circumstances, which has an increasingly important role in revealing the basic activities and laws of life (Li X. et al., 2021).

In this study, a 3-year phytoremediation experiment was conducted at different soil degradation levels to investigate how *L. chinensis* remediates the degraded soil environment by altering root exudate components and metabolism pathways. We evaluated the enhancement effect of root exudates on degraded soil nutrients. Root exudate component changes were detected by untargeted metabolomics based on gas chromatography–time-of-flight mass spectrometry (GC–TOFMS). Metabolic pathway analysis during the restoration process was performed according to the identified differential metabolic markers. In addition, the effects on physiological activities in root tissues were also analyzed. The obtained research findings provide new insights for defining the phytoremediation mechanism in degraded soils.

## Materials and Methods

### Experimental Area and Setup

**FIGURE 1:**
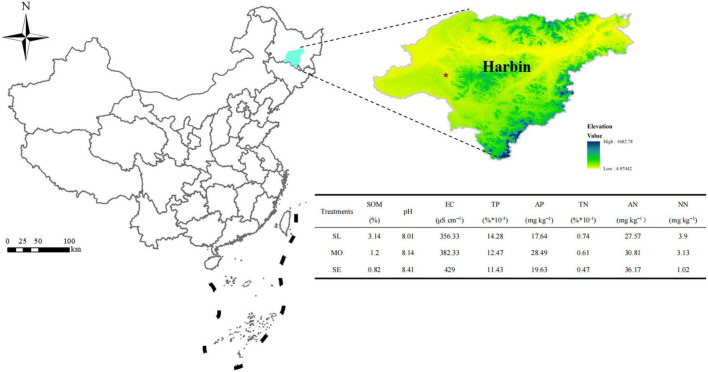
Location of the experimental area.

The experimental area was located at the demonstration base (126°26′04′′E, 45°57′87′′N) around Harbin, China (Figure 1). This site experiences a humid temperate continental monsoon climate, with an average annual precipitation of 350 mm and an annual average temperature of −5 to 4°C. More than 80% of the total precipitation occurs during the growing period, and the highest average temperature is 23°C. According to the land use survey results, three typical soil degradation levels were selected for experimental sites: slightly (SL), moderately (MO), and severely (SE) degraded soils. *L. chinensis* was planted on 20 July 2017. Each site used a land area of 5 m × 5 m, and the adjacent plot was untreated as a buffer to minimize the crossover effect. Each treatment was performed in triplicate according to a randomized block design.

### Effects of Root Exudate Release on Soil Remediation

The soil samples were harvested on 3 September 2020. The root systems in different degraded plots were collected randomly and shaken gently to remove the loose soil. The closely attached soil on the root surface (<3 mm from the root surface) was collected and defined as the rhizosphere soil (the three rhizosphere soils were marked as RSL, RMO, and RSE), and the soil without plants was bulk soil (Solá et al., 2019). The soil samples were mixed evenly, air-dried at 25°C, and passed through a 2-mm sieve to determine the soil’s physical and chemical properties. EC meters (Rex) and pH meters (Rex) were used to measure the electrical conductivity (EC) and pH, respectively (1:5 soil/water ratio, m/V). The potassium dichromate method was used to analyze SOM (Bao, 2010). Soil total phosphorus (TP), total nitrogen (TN), available phosphorus (AP), ammonia nitrogen (AN), and nitrate nitrogen (NN) were all detected by a continuous segmented flow analyzer (SEAL AutoAnalyzer 3 HR).

### Physiological Activities of Root Samples

The physiological activities of root samples were determined by malondialdehyde (MDA), proline (Pro), enzyme activity (POD), superoxide dismutase (SOD), catalase (CAT), and protein (Nanjing Jiancheng Bioengineering Institute, Inc., China) kits. MDA, Pro, POD, SOD, and CAT test kits were provided by Suzhou Keming Biotechnology Co., Ltd. (Suzhou, China). Fresh root samples (0.5 g) were homogenized with normal saline at a ratio of 1:9 w/v under low-temperature conditions and then centrifuged at 3,500 rpm/min for 10 min to obtain the supernatant liquid, and the sample absorbances of MDA, protein, POD, SOD, and CAT were detected at 532, 595, 420, 550, and 240 nm according to the test kit instructions. Root tissue (0.5 g) was shaken at 96°C for 10 min, and the supernatant was extracted according to the instructions. Then, the absorbance of Pro was read at 520 nm.

### Root Exudate Sample Collection and Detection

The roots were carefully collected, and any attached soil was carefully removed with Milli-Q water. Then, the roots were transferred to a glass conical flask containing 200 mL sterilized Milli-Q water and covered with aluminum foil to protect the roots from light. The plants were removed after 2 h to collect the liquid, and the process was repeated until the required amount of root exudates was obtained and filtered with glass fiber filters. Finally, a 10-mL methanol and dichloromethane mixture (50:50) was used to elute the SPE cartridges for the next step of derivatization (Lin et al., 2019). Forty milliliters of root solution was freeze-dried and transferred to 2-mL Eppendorf tubes, and 1,000 μL precooled extraction solution was added. Then, 10 μL ribitol (adonitol, 0.5 mg/mL stock) was added as the internal standard and vortexed for 30 s to mix the solution well. The samples were homogenized at 35 Hz for 4 min in a grinder, posttreated in ice water for 5 min, and then centrifuged at 12,000 rpm at 4°C for 15 min. The supernatant was pipetted into 1.5-mL Eppendorf tubes and dried. At this stage, 100 μL supernatant of each sample was mixed as a quality control sample (QC). Then, 20 μL methoxyamine hydrochloride (in pyridine 20 mg mL^–1^) was added and incubated for 30 min at 80°C, followed by derivatization with 30 μL BSTFA reagent (1% TMCS, v/v) for 1.5 h at 70°C. After cooling to room temperature, 5 μL of FAMEs (in chloroform) was added to complete the derivatization process for analysis.

### Metabolite Analytical Methodology

An Agilent 7890 gas chromatograph coupled with a Pegasus HT time-of-flight mass spectrometer was used to analyze root exudate-derived extracts, and a DB-5MS capillary column (30 m × 250 μm × 0.25 μm, J&W Scientific, Folsom, CA, United States) was used for separation. Then, 1 μL of the derivatized sample was injected in split mode, the purge flow rate of helium was 3 mL min^–1^, and the gas flow rate passing through the column was 1 mL min^–1^. The GC oven column temperature was set as 50°C for 1 min, increased to 310°C at a rate of 10°C min^–1^, and finally held for 8 min. The TOFMS injection, transfer line, and ion source temperatures were set as 280, 280, and 250°C, respectively. Ionization was achieved by a −70 eV collision energy electron collision. After a solvent delay of 6.30 min, the mass spectrometry data were collected in full-scan mode with an m/z range of 50–500, and TOFMS data were acquired at a rate of 12.5 spectra per second. The quality of the data was checked, and the deviation value was filtered to remove noise by the single data interquartile range method. Single peaks were filtered, and the low-mass ions that were more than 50% missing in the QC sample and more than 80% missing in the actual sample were removed. The data were normalized by the internal standard method based on the total ion current of each sample, and the missing values in the original data were simulated and filled using the minimum-half method. More than 30% of the relatively fluctuating relative standard deviation (RSD) ions with large fluctuations were filtered out in all QC samples.

### Data Analysis

Raw peak extraction, baseline correction, deconvolution analysis, peak integration, and alignment analysis of mass spectrometry data were allowed by the Chroma TOF package (V 4.3x, LECO). The calculated variable important for the projection (VIP) of the first principal component was used to measure the impact strength of differences between groups, and VIP scores over 1.0 were selected for further interpretation of metabolic markers. Finally, Student’s *t*-test (*P* < 0.05) was used to evaluate and filter the remaining variables. The mass spectra and retention times of the differential metabolic markers were matched by searching the LECO-Fiehn Rtx5 database. Finally, based on the Kyoto Encyclopedia of Genes and Genomes (KEGG) metabolic database, the metabolic pathway was constructed. The soil physical and chemical property data and physiological data of plant tissues were analyzed by Origin Pro 9.0 and SPSS 19.0. In addition, one-way ANOVAs followed by Duncan’s multiple range tests (*P* ≤ 0.05) were performed to check for differences in data.

## Results

### *Leymus chinensis* Root Exudates Significantly Improved Soil Nutrients

**FIGURE 2:**
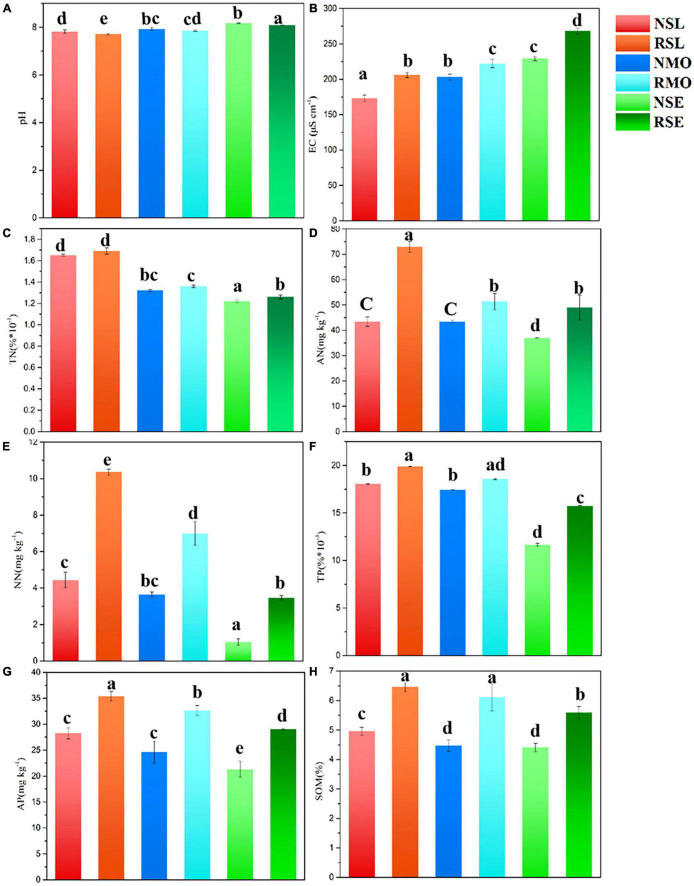
Effect of 3 years of phytoremediation on soil physical and chemical parameters, including pH **(A)**, EC **(B)**, TN **(C)**, AN **(D)**, NN **(E)**, TP **(F)**, AP **(G)**, and SOM **(H)**. The bars with different letters are significantly different (*P* < 0.05), and the results were obtained from one-way ANOVA. The first letter of the sample ID indicates the root compartment (R, rhizosphere soil; N, bulk soil).

According to our data (Figure 2), the SOM contents in SL, MO, and SE increased by 1.82, 3.27, and 3.59%, respectively, in a 3-year phytoremediation; due to root exudates, the contents increased by 1.50, 1.64, and 1.19% compared with the bulk soil. Phytoremediation increased the N content significantly, and root exudates also increased AN in RSL, RMO, and RSE by 40.52, 15.30, and 24.46%, NN by 57.14, 47.93, and 70.03%, and TN by 2.37, 2.94, and 3.17%, respectively, compared with bulk soil. There was a certain difference between the P and N contents; phytoremediation increased the TP content slightly, but the AP content increased significantly, and the TP content in the rhizosphere soil increased by 30.38, 34.75, and 24.62%, respectively. The pH values of RSL, RMO, and RSE were reduced by 0.24, 0.39, and 0.25 U, respectively, and EC levels were reduced by 15.92, 8.49, and 14.61%, respectively. The above results indicated that 3 years of phytoremediation improved the degraded soil nutrients well, indicating that the interaction between root exudates and soil cannot be ignored.

### Effects of Plant Physiological Activities

**FIGURE 3:**
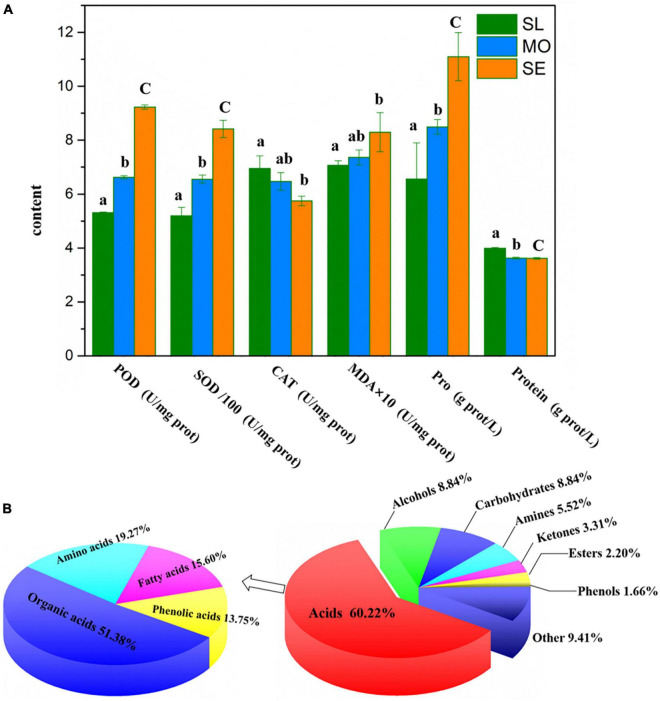
**(A)** Effect of soil degradation on physiological activities in plant tissues. The bars of different letters are significantly different (*P* < 0.05). **(B)** Classification of known metabolites and acids.

We analyzed the activities of POD, SOD, and CAT, the three important enzymes of root tissues, in different degraded soil levels due to the crucial role of antioxidant enzymes in ROS removal. As shown in Figure 3A, the activities of all three enzymes showed increasing trends with increasing soil degradation level, and the SE root system showed 1.74-, 1.62-, and 1.21-fold higher POD, SOD, and CAT activities than SL and MO, respectively. These results suggest that *L. chinensis* enhanced the activities of antioxidant enzymes to maintain a low level of ROS to cope with soil degradation by scavenging excess H_2_O_2_ and O_2_ in roots. The MDA level in H was 0.83 U/mg prot, which was enhanced by 14.78% compared with SL, but there was no significant difference between MO and SL. The levels of Pro, MO, and SE were enhanced by 22.82 and 31.31%, respectively, compared with those in SL roots, but there was no significant difference between MO and SE. In addition, the protein level in root tissues of SE and MO was significantly decreased compared with SL. These findings indicated that *L. chinensis* could adapt to soil degradation by changing the osmotic pressure of root cells, the cell membrane fluidity, and the protein content in plant tissues.

### Analysis of the *Leymus chinensis* Root Exudate Composition

We identified the main components of root exudates of *L. chinensis* based on GC–TOFMS metabolic profiling, which is a sensitive analysis. The overall pattern of metabolite changes after exposure to stressors can be monitored and has gradually been applied to study plant tolerance to abiotic stress. The GC–TOFMS molecular characteristic peaks were compared based on the HDMB, PubChem, and METLIN databases by retention time and similarity. The *L. chinensis* root exudate composition was complex, with a total of 473 peaks detected, of which 293 were unknown metabolites and 180 were named metabolites. The exudate contained 60.22% acids, alcohols, and carbohydrates, each 8.84%, with amines 5.52%, ketones 3.31%, esters 2.20%, and phenols 1.66%; metabolites <1.10% were classified as other substances, which comprised a total quantity of 9.41% (Figure 3B). Of the most important acids in root exudates, organic acids had the most types, accounting for 51.38%, followed by amino acids, fatty acids, and phenolic acids, accounting for 19.27, 15.60, and 13.75%, respectively (Figure 3B). In addition, *L. chinensis* root exudates also contain a variety of secondary metabolites, such as pyrimidines, pyridines, purines, terpenes, and diterpenes, which have been commonly detected in most plant root exudates, and some allelochemicals, such as benzoic acid and hydrocarbons.

### Changes in the *Leymus chinensis* Root Exudates Upon Growth With Soil Degradation Level

**FIGURE 4:**
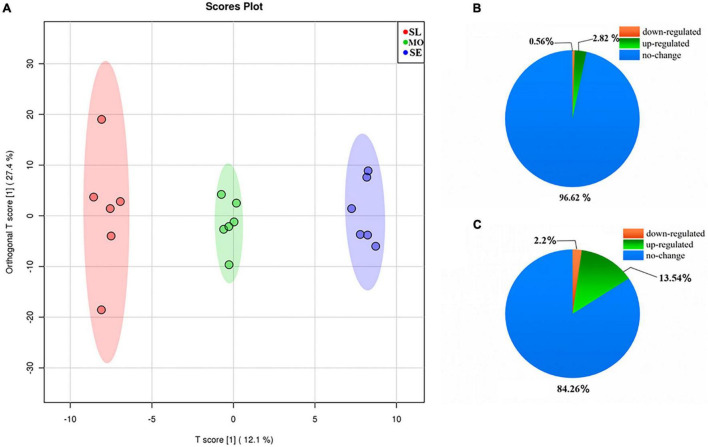
**(A)** OPLS-DA analysis of soil samples at different degradation levels. Percentages of MS peaks with no change, upregulation, and downregulation in SL/MO **(B)** and MO/SE **(C)**.

OPLS-DA was used to compare the influence of degraded soil on root exudates to distinguish metabolic changes under slightly, moderately, and severely degraded soil levels. OPLS-DA (Figure 4A) within the 95% confidence interval (Hotelling’s T-squared ellipse) showed that the sample points in the same treatment clearly clustered and showed small differences. A clear separation was observed in the MO degradation level from SL along PC1, and SE, with more severe degradation, was significantly separated from the MO and SL degradation levels along PC1. These results indicated that the soil degradation markedly changed the *L. chinensis* root exudates, and its components were dependent on the degradation level, indicating that it might adapt and restore different degradation levels in soil by adjusting its own metabolic system.

**FIGURE 5:**
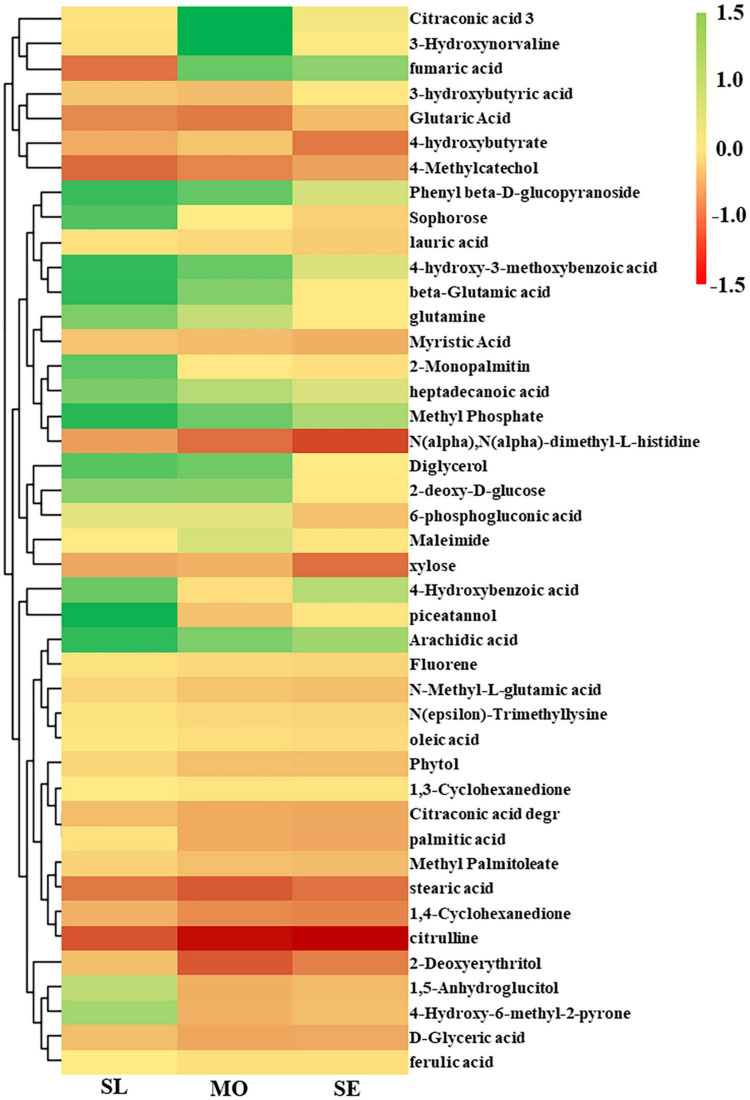
Heatmap analysis for the identified *L. chinensis* metabolites with significant differences (VIP > 1.0, *P* ≤ 0.05).

The differential metabolites of each treatment were determined by selecting MS peaks with Student’s *t*-test (*P* ≤ 0.05) and VIP scores. A total of 0.56 and 2.82% of the total MS peaks in the MO level group were significantly downregulated and upregulated, respectively, in comparison with the SL group (Figure 4B). In the SE degradation group, in comparison with the MO group, 2.20 and 13.54% of the total MS peaks were significantly downregulated and upregulated, respectively (Figure 4C). The numbers of upregulated and downregulated MS peaks increased with increasing soil degradation level. A total of 43 significantly different metabolites in comparison were identified according to the HDMB, PubChem, and METLIN databases, and hierarchical cluster analysis was used to visualize the relationships between these metabolites (Figure 5). The differential metabolites in root exudates included eight fatty acids, seven organic acids, six amino acids, three phenolic acids, four alcohols, three esters, three ketones, three sugars, two phenols, two amines, one fluorene, and one nucleoside. Two metabolites were downregulated, and 10 metabolites were upregulated in the comparison of the B and A groups. Among these identified compounds, eight and 49 metabolites were downregulated and upregulated, and 12 metabolites were upregulated and downregulated in the comparison between Group C and Group B. In summary, the above results indicated that the differential expression of primary and secondary metabolites in root exudates might be one of the reasons for plants adapting to or changing degraded soil.

### Combination of Metabolites in Root Exudates and Biological Endpoints

**FIGURE 6:**
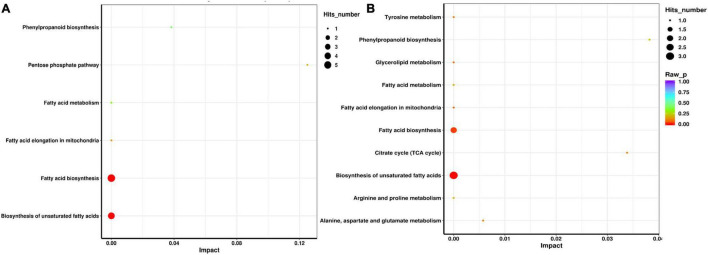
Pathway analysis of identified metabolites with significant differences in SL/MO **(A)** and MO/SE **(B)**.

The metabolites with significant differences were used to check the changes in metabolic pathways in the KEGG database. The enrichment pathway analysis revealed that there were six significantly enriched metabolic pathways in the MO/SL group (*P* < 0.05). In detail, these pathways included the pentose phosphate (ath00030), fatty acid biosynthesis (ath00061), fatty acid elongation (ath00062), fatty acid degradation (ath00071), phenylpropanoid biosynthesis (ath00940), and unsaturated fatty acid biosynthesis (ath01040) metabolic pathways (Figure 6A). The SE/SL group showed 10 significantly enriched metabolic pathways (*P* < 0.05). Figure 6B shows the citric acid cycle (TCA cycle) (ath00020), fatty acid biosynthesis (ath00061), fatty acid elongation (ath00062), fatty acid degradation (ath00071), phenylalanine, alanine, aspartate, and glutamate (ath00250), arginine and proline metabolism (ath00330), tyrosine metabolism (ath00350), glycerolipid metabolism (ath00561), phenylpropanoid biosynthesis (ath00940), and unsaturated fatty acid biosynthesis (ath01040). Taken together, the above results indicated that the abnormal expression of these primary and secondary metabolites by roots might be one of the reasons for plant adaptation or remediation of degraded soil.

**FIGURE 7:**
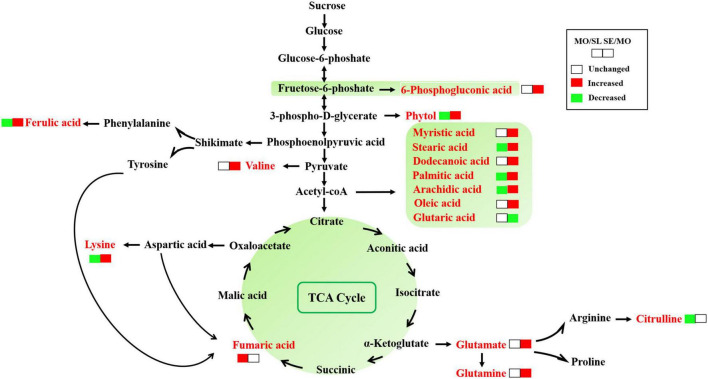
Alteration of related metabolic pathways of *L. chinensis* root exudates to adapt to soil degradation. The metabolites in red text are the differential metabolites. The red, green, and boxes indicate the metabolites with upregulation, downregulation, and no change, respectively.

The different metabolites of *L. chinensis* root exudates in different degraded soil levels were mapped to plant metabolic pathways, and the results are shown in Figure 7. In the primary metabolic networks, carbohydrates, amino acids, and lipid metabolites were usually significantly upregulated in the MO/SL and SE/SL groups. Many expression patterns were the same in the two groups. For example, metabolites such as lauric acid, stearic acid, oleic acid, and palmitic acid showed significant upregulation trends in lipid biosynthesis and metabolism-related pathways compared with the SL group. *L. chinensis* adapted to different degraded levels of soil environments by mobilizing different pathways in energy metabolism-related pathways. For example, it mobilized the pentose phosphate pathway (PPP) to adapt to the rhizosphere soil environment by upregulating the expression level of 6-phosphogluconate in MO/SL. Severely degraded soils upregulated the expression level of fumaric acid to mobilize the TCA cycle and amino acid biosynthesis and amino acid biosynthesis metabolic pathways *via* metabolite expression levels, such as glutamine, manganese–trimethyllysine, and ferulate, to adapt to the soil environment.

## Discussion

Soil quality is the primary determinant of food production or regional ecosystem resource regulation in the agricultural value chain, and a healthy soil environment is the key to human survival (Greiner et al., 2017). Food problems caused by unreasonable development and overutilization of agricultural soils around the world not only cause catastrophic economic losses for farmers and agricultural development restrictions but also reduce soil productivity continuously and even lead to soil desertification, which severely threatens ecological security (Ronchi et al., 2019; Kik et al., 2021). Currently, different soil remediation methods promote soil quality in countries around the world to maintain long-term soil sustainability (FAO, 2017). Phytoremediation, as a promising environmentally friendly soil remediation technology, has become a hot research topic for scholars worldwide. Non-food crop forage is not only simple field management but also provides strong support for animal husbandry and generates considerable economic benefits. Phytoremediation can fix carbon by photosynthesis, rhizodeposition below-ground, and litter materials above-ground to ensure the long-term sustainability of soil quality (Rasse et al., 2005). Root litters and plant shoots have been well studied, though living roots such as root exudates are still less well known.

Recent studies have shown that bioactive compounds released from root systems, namely, root exudates, play important roles in soil improvement and restoration issues (Xiong et al., 2019). Plants can adjust and even improve the environment by adjusting the main components of root exudates when confronted with external adverse factors. In this research, we investigated the rhizosphere ecological mechanism of *L. chinensis* phytoremediation of degraded soil by analyzing root tissue physiological activities and the root exudate metabolic profile. ROS have emerged as the main signaling molecules in plants in response to stress conditions (Chen and Yang, 2020). Soil degradation induces the accumulation of ROS in plants and gradually destroys cell lipids, proteins, and DNA. Then, plants mobilize complex antioxidant defense systems to synthesize antioxidant substances and enhance antioxidant enzyme activities to maintain the intracellular ROS metabolic balance and resist the plant cell oxidative damage suffered by degraded soil environments. POD, SOD, and CAT are the most important antioxidant enzymes in this process and coordinate with each other (Hu et al., 2021). SOD converts superoxide anions into H_2_O_2_ *via* disproportionation; subsequently, POD and CAT convert H_2_O_2_ into H_2_O and O_2_. The activities of SOD, POD, and CAT in plant root tissues seem to increase with the soil degradation level, indicating that ROS metabolism might play an important role in the resistance of *L. chinensis* to soil degradation and that plants mobilize antioxidant POD to remove excess ROS. MDA is produced in the lipid peroxidation process caused by free radical oxidation and is an important indicator of lipid peroxidation (Sadauskiene et al., 2018). The MDA contents in SL and SE plant root tissues seemed to increase with increasing soil degradation levels, indicating that *L. chinensis* might respond to soil degradation by increasing membrane lipid peroxidation. However, the difference in MDA between SE and MO was not significant, which might indicate that it has a strong resistance to soil degradation. Protein content was related to plant growth, with the lowest content in SE, indicating that soil degradation had a certain negative impact on protein synthesis (Gao et al., 2019). The above data indicated that *L. chinensis* might adapt to degraded soil environments by mobilizing different physiological activities.

The *L. chinensis* root exudates were complex, and the main components were acids, alcohols, carbohydrates, and other primary metabolites, which have been commonly detected in most plant root exudates (Zhang et al., 2014; Wu et al., 2015). Acid had great effects on soil formation and nutrient and pollutant conversion, and the root system could adjust these substances to cope with differences in hydraulic conditions and nutrient concentrations. A previous study showed that plants increased organic acid exudation under P-limited conditions (Edayilam et al., 2018). This was due to organic acids lowering the rhizosphere soil pH, mobilizing the activity of P, K, Ca, and other soluble mineral nutrients in the rhizosphere, and enhancing the absorption rate of plant nutrients. This was confirmed by our results showing that the rhizosphere soil pH was lower than that of bulk soil and that the contents of AP, AN, and NN in rhizosphere soil were also significantly higher than in bulk soil. The presence of acetic acid could also increase the accessibility of microorganisms to mineral-protected SOM by a non-biological dissolution reaction (Keiluweit et al., 2015). Root exudates provide vital nutrients and energy substances for soil microorganism growth and reproduction and have a selective effect on the microbial community, which has a certain ability to shape the rhizosphere microbiota. The presence of citric acid might increase the number of functional bacteria and improve the microbial community structure (Ma et al., 2020). Malic acid promoted the growth and colonization of *Paenibacillus polymyxa* and *Bacillus amyloliquefaciens*, which induce plant systemic resistance (Martins et al., 2018). Succinic acid and oxalic acid are considered to be the main C sources of denitrification microorganisms (Liu et al., 2016), and some phenolic substances have been shown to promote organic matter in soil decomposition by stimulating changes in microbial community composition (Huang et al., 2019). Plant roots can release amino acids and exert a variety of biological effects by passive diffusion; for example, the osmotic adjustment substance proline can protect cell membranes from oxidative stresses and stabilize protein structure (Li et al., 2019). In addition, some substances such as benzoic acid, hydrocarbons, and others detected were allelopathic substances, which could affect seed germination and microbial growth and possibly interfere with the growth of other plants. In addition to the primary metabolites, several secondary metabolites were detected, including atropine, polyphenols (cinnamic acid), terpenes, and diterpenes such as cuminic alcohol and phytol, ethanolamine, and 1,3-propylene diamine, which might be beneficial for slow-growing microorganisms that rely on low concentrations of secondary plant metabolites.

The release of root exudates is a dynamic process and may be greatly influenced by various environmental stress factors; it affects not only the soil ecosystem structure and resilience but also plant–soil feedback and long-term ecosystem maintenance, soil function, and stability (including nitrification) (Williams and de Vries, 2020; Li Y. et al., 2021). Plant metabolites are diverse and have complex metabolic pathways; therefore, differential metabolite detection and metabolic pathway analysis may reflect the state during plant growth. In our data, the contents of most fatty acids in root exudates were upregulated under exposure to the degraded soil, and *L. chinensis* mobilized fatty acid metabolism to promote the lipid composition in root cells and the adjustment of the membrane fatty acids. Lipids are the most compliant biomolecules; they are important parts of biofilms and protect cells from oxidative stress responses (Zhou et al., 2012). As a sensory device for contact with external signals, cell membranes mediate various reactions caused by external factors to cells, which is the target of harmful components of toxicity in the degrading soil. ROS in degraded soil attack root cells, and to adapt to the pressure of external factors, they increase saturated unsaturated fatty acid metabolism, such as palmitic acid, stearic acid, and arachidic acid, to maintain the fluidity and stability of biofilms and protect plant cell membranes from reactive oxygen species attack, which is also consistent with the physiological and biochemical indicator results (Wang et al., 2019). Therefore, the increase in fatty acid levels in root exudates in degraded soil may be an indicator of the root membrane resisting external environmental pressure. Zhao et al. (2018) also observed increased levels of fatty acid content in cucumber leaves under low copper stress, which suggested that it helps maintain the fluidity necessary for proper membrane function. Jung et al. (2015) also found that unsaturated fatty acids increased when burdock roots were exposed to copper. In short, degraded grassland might change the root exudate composition by upregulating fatty acid metabolism pathways of *L. chinensis* and changing the fluidity of root cell membranes.

Our results showed that 6-phosphogluconic acid was significantly affected by slightly degraded soils, indicating that the glucose produced by *L. chinensis* was phosphorylated rapidly and fueled the PPP preferentially. The PPP communicates with lipid, protein, nucleic acid, and secondary biomass metabolism. It provides building blocks for nucleotide biosynthesis and reducing power. It can also be routed to glycolysis intermediates to generate ATP, which provides the material basis for plant growth (de Tredern et al., 2021). A main function of the PPP is to produce NADPH, which is necessary to participate in photosynthesis and readily relieves the oxidative stress induced by ROS that provokes cell damage (Bolaños and Almeida, 2010). Therefore, *L. chinensis* supported the participation of non-oxidative glucose consumption to regulate antioxidant levels in the slightly degraded soil and, thus, the ROS concentration within rhizosphere cells. The influence of severely degraded soil on energy metabolism was more inclined to the TCA cycle. We found that intermediate metabolites such as citric acid and fumaric acid in the TCA cycle were upregulated. The TCA cycle provides substrates and energy for the synthesis of other metabolites and is a major part of plant aerobic respiration (Niehaus, 2021). For severely degraded soil, the results indicated that the TCA cycle pathway was greatly affected, and *L. chinensis* may adjust root cell respiration metabolism to adapt to severely degraded soil, which was confirmed by metabolic pathway analysis (Figure 7). In addition, severe degradation data showed that the metabolism of a variety of amino acids was clearly affected, indicating that *L. chinensis* also mobilizes protein breakdown to adapt to stress. Protein interactions are the basis of cells in the plant growth and development process, and protein interactions can produce a variety of effects. Thus, it can be considered that severely degraded soil environmental pressure has a certain role in promoting photosynthesis and maintaining energy balance.

## Conclusion

This investigation is a preliminary observation of the role of *L. chinensis* root exudates in the soil remediation process. The results showed that root exudates significantly improved soil nutrients, and the root cell tissue also mobilized oxidative stress to respond to the soil environment pressure. Some osmotic regulators and defense-related metabolites were significantly upregulated in root exudates to remediate degraded soils, and they mainly mobilized a series of fatty acid metabolic pathways to maintain the fluidity for membrane function to help plants adapt to adverse conditions. The PPP and TCA energy metabolism pathways were mobilized in response to slight and severe degradation environmental pressures, respectively. This study defines *L. chinensis* metabolic pathways and root exudate components related to the degraded soil response and offers valuable insights into the phytoremediation mechanism in degraded soils.

## Data Availability Statement

The raw data supporting the conclusions of this article will be made available by the authors, without undue reservation.

## Author Contributions

YL and PZ performed the data analyses and wrote the manuscript. QWu and YZ contributed significantly to analysis and manuscript preparation. QWe helped to perform the analysis with constructive discussions. YS and SS performed the experiment. GC contributed to the conception of the study. All authors contributed to the article and approved the submitted version.

## Conflict of Interest

The authors declare that the research was conducted in the absence of any commercial or financial relationships that could be construed as a potential conflict of interest.

## Publisher’s Note

All claims expressed in this article are solely those of the authors and do not necessarily represent those of their affiliated organizations, or those of the publisher, the editors and the reviewers. Any product that may be evaluated in this article, or claim that may be made by its manufacturer, is not guaranteed or endorsed by the publisher.
